# Kernel-wise difference minimization for convolutional neural network compression in metaverse

**DOI:** 10.3389/fdata.2023.1200382

**Published:** 2023-08-04

**Authors:** Yi-Ting Chang

**Affiliations:** Department of Computer Science, National Tsing Hua University, Hsinchu, Taiwan

**Keywords:** metaverse, computer vision, Huffman coding, filter-level pruning, CNN

## Abstract

Convolutional neural networks have achieved remarkable success in computer vision research. However, to further improve their performance, network models have become increasingly complex and require more memory and computational resources. As a result, model compression has become an essential area of research in recent years. In this study, we focus on the best-case scenario for Huffman coding, which involves data with lower entropy. Building on this concept, we formulate a compression with a filter-wise difference minimization problem and propose a novel algorithm to solve it. Our approach involves filter-level pruning, followed by minimizing the difference between filters. Additionally, we perform filter permutation to further enhance compression. Our proposed algorithm achieves a compression rate of 94× on Lenet-5 and 50× on VGG16. The results demonstrate the effectiveness of our method in significantly reducing the size of deep neural networks while maintaining a high level of accuracy. We believe that our approach holds great promise in advancing the field of model compression and can benefit various applications that require efficient neural network models. Overall, this study provides important insights and contributions toward addressing the challenges of model compression in deep neural networks.

## 1. Introduction

Deep neural networks, especially deep convolutional neural networks, have achieved remarkable success in computer vision tasks. However, to pursue better performance, many convolutional neural network architectures (Simonyan and Zisserman, 2014; Szegedy et al., 2015; He et al., 2016; Krizhevsky et al., 2017) have been designed. These CNNs with high accuracy, however, tend to be heavy and consist of multiple convolution layers with a large number of parameters (e.g., 248 MB for AlexNet and 552 MB for VGG16). Moreover, many applications today demand good performance on mobile devices or embedded platforms with limited storage space and computation power, making it challenging to utilize heavy CNNs on these devices. Therefore, model compression has emerged as a popular research topic in recent years. Various approaches, such as parameter pruning (Han et al., 2015; Li et al., 2016; Wen et al., 2016; Luo et al., 2017; Huang et al., 2018), parameter quantization (Gupta et al., 2015; Courbariaux et al., 2016; Umuroglu et al., 2017), low-rank factorization (Lebedev et al., 2014; Kim et al., 2015), and knowledge distillation (Romero et al., 2014; Hinton et al., 2015; Yim et al., 2017), have been proposed to reduce the model size and computation costs while maintaining acceptable inference accuracy. However, the authors of Han et al. (2015) did not consider the best-case scenario for model compression using Huffman coding as we know that data with lower entropy can be compressed more efficiently using Huffman coding. Therefore, our proposed algorithm is designed based on this concept. Although it is not possible to directly change the parameters into low entropy distribution, as this could harm the model's accuracy, we employ delta coding on filters to transform the parameter distribution for Huffman coding. To this end, we formulate a new problem for model compression, named compression with filter-wise difference minimization (CFDM), which aims to minimize the differences between filters while maintaining acceptable accuracy. This approach benefits from Huffman coding with delta coding to further compress the model. To solve this problem, we propose an algorithm that performs clustering on filters and applies a new penalty function to minimize the difference between filters in a cluster. Furthermore, we apply filter permutation to lower the entropy of delta-coded parameters and achieve further compression. Our proposed algorithm achieves a compression rate of 50× for Lenet5 and 94× for VGG16, significantly outperforming the state-of-the-art approach (Han et al., 2015). Overall, this study contributes to the field of model compression by proposing a novel algorithm that efficiently reduces the size of deep neural networks while maintaining acceptable accuracy. The proposed approach offers promising results and can benefit various applications that require efficient neural network models with limited resources.

The contributions of this study are summarized as follows.

1. We consider the best situation of Huffman coding, and we propose a new problem, named compression with filter-wise difference minimization (CFDM).
2. We propose an algorithm for convolution layer compression, which considers the best situation for Huffman coding, then train the model with a proposed penalty function. In addition, we apply filter permutation for further compression.

## 2. Related work

### 2.1. Compression in big neural network

Neural network compression has become an important area of research as it enables models to run faster by reducing their size and computational requirements. This is particularly useful for applications that require fast model performance (Yang et al., 2021; Chang et al., 2022; Yang and Shen, 2022). Recent neural network compression approaches can be broadly classified into four categories: parameter pruning, parameter quantization, low-rank factorization, and knowledge distillation. Parameter pruning approaches (Han et al., 2015; Li et al., 2016; Wen et al., 2016; Luo et al., 2017; Huang et al., 2018) are dedicated to significantly reducing the model size with an acceptable accuracy loss by removing redundant parameters and fine-tuning. Various approaches have been proposed to select the redundant parameters, such as iterative pruning methods (Han et al., 2015) and structurally pruning convolutional layers (Li et al., 2016; Wen et al., 2016; Luo et al., 2017; Huang et al., 2018; Tung and Mori, 2018). For example, structured sparsity learning (Wen et al., 2016) uses proposed regularizers to learn structured sparsity, while filter-level pruning (Li et al., 2016) selects redundant kernels by calculating the L1-norm of each filter. In addition, some methods (Luo et al., 2017) utilize information from the next layer to measure the importance of filters. Parameter quantization approaches (Gupta et al., 2015; Courbariaux et al., 2016; Umuroglu et al., 2017; Tung and Mori, 2018; Jiao et al., 2021; Tonin and de Queiroz, 2022; Xu et al., 2023) aim to reduce the number of bits used to represent each parameter to save memory. Early works such as Kmeans clustering (Han et al., 2015) and HashedNet (Chen et al., 2015) have been proposed to quantize each parameter. HashedNet utilizes a hash function to group parameters into hash buckets for quantization. The optimal quantization bit-width of each layer can be found through optimal bit allocation (Chen et al., 2015). Deep compression (Han et al., 2015) combines pruning and quantization and then encodes the quantized parameter by Huffman coding for further compression. Additionally, diagonal block-wise difference minimization (Hsu et al., 2020) proposes a novel method for compressing neural networks by minimizing diagonal block-wise differences. HPTQ (Xu et al., 2023) proposes a method for super-resolution neural networks that integrates layer-wise quantization and piece-wise quantization based on error sensitivity and the quantization error of parameters to reduce the storage cost of these networks. Tonin and de Queiroz (2022) discussed the quantization of neural networks for compression and representation without retraining, aiming to facilitate their deployment in standard formats. Jiao et al. (2021) presented a technique called synchronous weight quantization-compression (SWQC) to compress the weights of low-bit quantized neural networks (QNNs). The SWQC technique quantizes the weights based on compression efficiency and their probabilities of having different quantized results, and with the help of retraining, a high compression rate and accuracy can be achieved. However, Xu et al. (2023) is predicated on the use of a super resolution (SR) neural network, primarily intended for image super-resolution applications. In contrast, our study examines neural networks that primarily consist of convolution layers. The structural differences between these two types of neural networks make the methodology from Xu et al. (2023) non-transferable to ours directly. The focus of Tonin and de Queiroz (2022) is on compression that avoids retraining and seeks to prevent any substantial changes to the model's architecture, such as the insertion or removal of layers. Contrarily, our study assumes a predetermined set of pruning ratios for each convolution layer and stipulates the utilization of filter clustering within each convolution layer. Tonin and de Queiroz (2022) underscored the importance of preserving the model's performance post-compression without explicitly defining the performance measure. Our study, however, sets a clear target of minimizing filter-wise disparity within each filter cluster after compression and promotes structural sparsity. The central differentiation between Jiao et al.'s (2021) study and our study lies in the underlying assumptions and employed methodologies. Jiao et al. (2021) presumes that weights can be quantized independently, and subsequent compression can be achieved via symmetric quantization and encoding methods. Conversely, our methodology posits that filters within the same convolutional layer can be clustered and that these clusters can be compressed by minimizing inter-filter discrepancies. As such, given the fundamental differences in the assumptions and methods of the two studies, the method of Jiao et al. (2021) cannot be straightforwardly applied to our study. In Jiao et al.'s (2021) study, compression transpires at the individual weight level, whereas in our study, compression occurs at the filter level. This distinction forms the primary demarcation between the two methodologies.

### 2.2. Application for social network in metaverse

The metaverse is a term used to describe a collective virtual shared space created by the convergence of the internet and the physical world. It is a place where users can interact with each other and with virtual objects in a shared virtual environment. Social networks play an important role in the metaverse, allowing users to connect and form communities within virtual worlds (Shen et al., 2011, 2017). One potential application of social networks in the metaverse is in organizing events and activities within virtual worlds. Yang et al. (2012) and Shen et al. (2015) proposed a socio-spatial group query (SSGQ) for location-based social networks that could be used to select a group of nearby attendees with tight social relationships for impromptu activities within a virtual world. Similarly, an Unfamiliarity-Aware Therapy Group Selection with Noah's Ark Principle (UTNA) could be used for automatic selections of therapy group members from the social network while addressing crucial criteria such as avoidance of isolation and loneliness, the unfamiliarity of patients, and size of the therapy group (Hsu et al., 2018). Social networks could also be used to organize online soirees with live multi-streaming within the metaverse. A social-aware diverse and preferred live streaming channel query (SDSQ) could jointly select a set of diverse and preferred live streaming channels and a group of socially tight viewers for an activity within a virtual world (Shen et al., 2018).

## 3. Problem description and observations

### 3.1. Problem formulation

The compression with filter-wise difference minimization (CFDM) problem is formulated as follows. Given a neural network with *L* convolution layers, a set of pruning ratios ℙ = {*p*_1_, …, *p*_*l*_, …, *p*_*n*_} for each convolution layer 1 ≤ *l* ≤ *L*, where *p*_*l*_% of parameters would be kept [i.e., (1 − *p*_*l*_)% of parameters should be pruned], the quantization bits *q*_*l*_ for parameters, and the cluster number *k* for each convolution layer, suppose the filter matrix of a convolution layer is presented by 4D tensors ${𝕎}^{l}=\left\{w_{1}^{l},w_{2}^{l},\ldots ,w_{i}^{l},\ldots ,w_{N_{l}}^{l}\right\}$, where $w_{i}^{l}\in {\mathbb{R}}^{M\times h\times w}$. Let *N*_*l*_ denote the number of filters in the *l*-th layer and *M, h, w* be the number of input feature maps, the height, and the width of the *l*-th layer kernel. The proposed method in this study will cluster filters of each convolution layer into *k* clusters. The goal of CFDM is to minimize model size with an accuracy drop in 1%, such that each convolution layer forms in structure-sparsity after pruning and the filter-wise difference in each filter cluster should be small.

### 3.2. Observations

**Figure 1:**
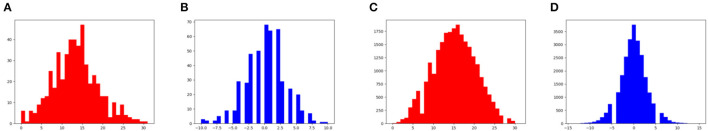
Lenet5 distribution, **(A)** is conv1. index, **(B)** is conv1. delta-coded, **(C)** is conv2. index, and **(D)** is conv2. delta-coded.

In this section, we introduce the observation that applying delta coding with Huffman coding on similar filters (minimum filter-wise differences) after quantization is the benefit to the model compression, which is the main concept in this study. After quantization, parameters are stored in only fewer index numbers (i.e., if *q* is 5, parameters are only stored in 0–31). Therefore, Han et al. (2015) applied Huffman coding for lossless further compression after quantization. However, Han et al. (2015) did not consider controlling the distribution of parameters to optimize the effectiveness of Huffman coding. As we know, Huffman coding could significantly compress the data when the data form in the bias distribution, that is, lower entropy of parameters could make better compression. According to this concept, we observed that generating similar filters, then applying filter-wise delta coding could generate the delta-coded parameters with lower entropy. Due to that when quantization on similar filters, more parameters tend to share the same indices, so delta coding on these filters could convert distribution into a more biased distribution. To validate the idea above, we did a simple preliminary experiment for Lenet5 on MNIST. There are two convolution layers in Lenet5 (called conv1 and conv2), and the corresponding number of filters are 20 and 50, doing compression with 5 bits quantization to each layer and then applying delta coding to convert filters into delta-coded parameters. For validating the minimum filter-wise differences benefits to model compression, we added the penalty term to the loss function to minimize the total differences of consecutive filter-pairs (i.e., suppose filters of conv1 represent as {*F*_1_, …, *F*_*i*_, …, *F*_20_}). The consecutive filter-pairs are {(*F*_1_, *F*_2_), …, (*F*_*i*_, *F*_*i*+1_), (*F*_19_, *F*_20_)}) during training. Table 1 shows the model size comparison of two kinds of models: model (H), the model trained by penalty function without delta coding (min. difference +H) and the model trained by penalty function with delta coding (min. difference +D+H). All of these models apply quantization and Huffman coding. Note that the further compression by delta coding is due to the distribution of the delta-coded parameters being more biased than the distribution of the original parameters, and the comparison is shown in Figure 1. To summarize this preliminary experiment, the idea of minimizing filter-wise differences could achieve better model compression.

**Table 1:** Preliminary result for minimizing filter-wise difference.

	**H**	**Min.difference+H**	**Min.difference+D+H**
Conv1	420B	430B	383B
Conv2	13747B	13571B	11252B

## 4. Proposed algorithm

In this section, we propose an algorithm for solving the compression with filter-wise difference minimization (CFDM) problem for compressing convolution layers. The proposed algorithm consists of the following steps: filter-level pruning, filter-wise differences minimization, quantization, filter permutation, delta coding, and Huffman coding. As we mentioned in Section 3.2, the filter-wise differences minimization benefits Huffman coding with delta coding as the delta-coded parameters have lower entropy. Therefore, the proposed algorithm flow is designed based on the observation in Section 3.2. Since most models have redundant parameters, pruning is necessary for an effective model compression approach. Thus, in the first step, we applied filter-level pruning to reduce the model size. In the second step, based on the observation of filter-wise difference minimization, we clustered the pre-trained filters into *k* filter clusters of each layer and then apply a new loss function to minimize the total differences between consecutive filter pairs within each cluster. In the third step, we applied *k*-means quantization as in Han et al. (2015) on each convolution layer and retrained the *k*-centroids until the model accuracy is recovered. In the fourth step, to further minimize the entropy of delta-coded parameters, we applied a permutation of filters in each filter-cluster to minimize the cyclic distance between consecutive filters. Finally, in the last step, we applied Huffman coding on the delta-coded matrix parameters, which are generated by doing delta coding on each reordered filter-cluster. In the following, we present the details of each step. Overall, the proposed algorithm takes advantage of multiple techniques to achieve effective model compression while preserving model accuracy.

### 4.1. Filter-level pruning

At the pruning step, it is needed to consider the pruning effect for the filter-wise difference minimization in the next step. For example, suppose that we apply the pruning method in Han et al. (2015) which pruned the parameters whose values are below the given threshold and the pruned parameters are directly assigned the value zero. However, these fixed and irregular zero parameters are a huge constraint for minimizing the filter-wise differences. It is hard to control the delta-coded parameter distribution on these irregular sparse parameters due to the fixed zero index value cannot be changed. Especially, recent studies such as Li et al. (2016), Wen et al. (2016), and Luo et al. (2017) focused on structure pruning on convolution layers, and some of them had applied channel-level pruning, which removes the redundant channels of filters and some had applied filter-level pruning which removes the filters and the corresponding output feature maps. Therefore, for not to affect the performance of the minimization of filter-wise difference, we applied the filter-level pruning method proposed by Li et al. (2016). We pruned the filters which are the top (1 − *p*_*l*_) percent smallest L1-norm $\left||w_{i}^{l}|\right|$ filters on the *l*-th convolution layer and then we retrained the model to recover the model accuracy.

### 4.2. Filter-wise difference minimization and quantization

In step 1, we pruned the filters of convolution layers not only for reducing the model size but also to avoid irregular sparse parameters, so that it does not exist the fixed zero issue which could affect the calculation of filter-wise difference. In step 2, the goal is to minimize the filter-wise difference with an acceptable accuracy drop. By doing so, in step 3, the minimum filter-wise difference is a good property for applying delta coding on quantized filters. First, recalling the preliminary experiment in Section 3.2, we added a penalty term to the loss function and minimized the differences of consecutive filter pairs during training. It seems that it is the most straightforward method for minimizing the differences between the filters. However, when applying the above method to the convolution layers with many filters, it will be hard to converge without an accuracy drop. In addition, there is an issue that as we know the filters are regarded as the patterns for convolutional neural networks, it is hard to make whole filters in a convolution layer similar without any accuracy drop, especially in the layer with many filters. Therefore, we proposed the algorithm, at first, we cluster filters into *k* clusters in which filters have similar patterns so that it could avoid the issue mentioned above. For filter clustering, we applied k-means to the remaining filters ${𝔽}^{l}=\left\{f_{1}^{l},\ldots ,f_{i}^{l},\ldots ,f_{r_{l}}^{l}\right\}$ of each convolution layer *l*, where 𝔽^*l*^∈𝕎^*l*^. Let $r_{l}=\left\lceil \frac{N_{l}\dot{\left(1-p_{l}\right)}}{100}\right\rceil$ denote the remaining number of filters of *l*-th convolution layer. Denote *k* filter-clusters as $\left\{{𝕊}_{1}^{l},\ldots ,{𝕊}_{i}^{l},\ldots ,{𝕊}_{k}^{l}\right\}$ and $S_{i}^{l}=\left\{s_{i1}^{l},\ldots ,s_{ij}^{l},\ldots ,s_{im_{i}^{l}}^{l}\right\}$, where $m_{i}^{l}$ is the number of filters of *i* filter-cluster in *l* layer. Note that $s_{ij}^{l}\in F^{l}$ and $\cup_{i}S_{i}^{l}=F^{l}$ and $\cap_{i}S_{i}^{l}=\emptyset$. The corresponding centroids which are generated by *k*-means are denoted as $\left\{c_{1}^{l},\ldots ,c_{i}^{l},\ldots ,c_{k}^{l}\right\}$ in each convolution layer. Then, we proposed the penalty function $L_{P}$ for minimizing the total differences of filters of each filter-cluster $S_{i}^{l}$, which is shown as follows:


(1) $${ℒ}_{P}=\frac{1}{L}\sum_{l=1}^{L}\frac{\sum_{i=1}^{k}\sum_{j=1}^{m_{i}^{l}}{\left\|s_{ij}^{l}-c_{i}^{l}\right\|}^{2}}{r_{l}}$$

The penalty function is designed to minimize the total difference between filters and their corresponding centroid. Thus, the penalty will lead to filters in a filter-cluster more similar. Based on $L_{P}$, for training within 1% accuracy drop, the loss function $L_{total}$ is designed as follows:


(2) $$\begin{array}{l} L_{total}=L_{acc}+\alpha L_{P}, \end{array}$$

where $L_{acc}$ is the accuracy loss which can be cross-entropy or *MSE* depending on the training task, and α is a hyper-parameter to control the trade-off penalty between the compression rate and the model accuracy. If large α value is applied, it means the training procedure is more dominant to minimize the differences between filters and their corresponding centroids. Therefore, it is more likely that parameters share the same index value in each filter-cluster after quantization. Thus, the larger α value will result in more compression but may lower the model accuracy. In contrast, a smaller α would be more likely to minimize the original error loss. Therefore, α is an important factor for compression rate, and there is an α sensitivity experiment in Section 5.3. The pseudocode for filter-wise difference minimization is listed in Algorithm 1. After the model converges, next, we applied *k*-means quantization in Han et al. (2015), quantizing filters $\left\{S_{1}^{l},\ldots ,S_{i}^{l},\ldots ,S_{K}^{l}\right\}$ according to the given *q*_*l*_ of each convolution layer *l* and then we retrained the centroids until model recovers the accuracy. Let the quantized filters which are stored in index value denote as $\left\{B_{1}^{l},\ldots ,B_{i}^{l},\ldots ,B_{K}^{l}\right\}$, where $B_{i}^{l}=\left\{b_{i1}^{l},\ldots ,b_{ij}^{l},\ldots ,b_{im_{i}^{l}}^{l}\right\}$ and $b_{ij}^{l}\in \mathbb{Z}=\left\{0,\ldots ,2^{q^{l}}-1\right\}$.

**Algorithm 1:** Filter-wise difference minimization.

**input:** Remaining filters of each layer: $\left\{F^{1}=\left\{f_{1}^{1},\ldots ,f_{r1}^{1}\right\},\ldots ,F^{l}=\left\{f_{1}^{l},\ldots ,f_{rl}^{l}\right\},\ldots ,F^{L}=\left\{f_{1}^{L},\ldots ,f_{rL}^{L}\right\}\right\}$, training epochs *k*
**function** K-means(*F*^*l*^)
**return** filter-clusters of *l* layer$\left\{S_{1}^{l},\ldots ,S_{K}^{l}\right\}$, cluster-centroids of *l* layer$\left\{c_{1}^{l},\ldots ,c_{K}^{l}\right\}$
**function** LP(filter-clusters, cluster-centroids)
$i\leftarrow 1,j\leftarrow 1,L_{p}\leftarrow 0$
**for** *i* ≤ *K* **do**
**for** $j\leq size\left(S_{i}^{l}\right)$ **do**
$L_{p}\leftarrow L_{p}+||s_{ij}^{l}-c_{i}^{l}||$
*j*←*j*+1
*i*←*i*+1
$L_{p}\leftarrow \frac{L_{p}}{r_{l}}$
*l*←*l*+1
**return** $\frac{L_{p}}{r_{l}}$
**for each** Remaining filters of each layer: *F*^*l*^ **do**
$\left\{S_{1}^{l},\ldots ,S_{K}^{l}\right\},\left\{c_{1}^{l},\ldots ,c_{K}^{l}\right\}\leftarrow$ K-means(*F*^*l*^)
filter-clusters ← filter-clusters $+\left\{S_{1}^{l},\ldots ,S_{K}^{l}\right\}$
cluster-centroids ← cluster-centroids $+\left\{c_{1}^{l},\ldots ,c_{K}^{l}\right\}$
**for each** training epoch **do**
Input batch data and calculate the *MSE*(or cross-entropy)
Loss ← *MSE*; Loss ← Loss + LP(filter-clusters, cluster-centroids)
Compute gradient, back propagation

### 4.3. Filter permutation with delta and Huffman coding

In this section, we proposed a penalty to minimize the differences between filters and their corresponding centroids in each filter-cluster, so that the filters in a filter-cluster will tend to be more similar. Thus, the filters in a filter-cluster will be more likely to share the same index value after quantization. Based on this property, in this section, we applied delta-coding to convert the filters into a first filter and delta-coded filters of each filter-cluster $B_{i}^{l}$, where 1 ≤ *i* ≤ *K* in the *l* layer. Here, we give an example to easily understand.

**Example 1**. Consider a scenario where we have three quantized filters in a filter-cluster. These filters are represented as *b*1, *b*2, and *b*3, where each filter is a 2× 2 matrix quantized to 3 bits. Specifically, the individual filters are defined as

*b*1 = [[0, 1], [2, 1]], *b*2 = [[7, 2], [7, 2]], and *b*3 = [[1, 3], [1, 3]] Our goal is to apply delta coding on these filters in a sequential order, taking into account their cyclic distance. The cyclic distance gives us the measure of difference between two successive filters in our given sequence.

In implementing delta coding, we first select *b*1 as the starting filter. Subsequently, we calculated the cyclic distance from *b*1 to *b*2, represented as |*b*1−*b*2|. This gives us a new 2× 2 matrix:

|*b*1−*b*2| = [[−1, 1], [5, 1]]. Next, we calculated the cyclic distance from *b*2 to *b*3, represented as |*b*2−*b*3|. This operation results in another 2× 2 matrix:

|*b*2−*b*3| = [[2, 1], [2, 1]]. Therefore, after applying delta coding with cyclic distances, our original sequence of filters *b*1, *b*2, and *b*3 transforms into the delta-coded sequence *b*1, |*b*1−*b*2|, and |*b*2−*b*3|. This new sequence effectively represents the relative differences between each consecutive pair of filters.

Note that the selection of *b*1 as the initial filter is not a hard-and-fast rule. Depending on the specific requirements or constraints of the problem, we could begin the delta coding process with any filter in the sequence.

In Example 1, note that ||*b*1−*b*2|| is delta-coded as [−1, 0] instead of [7, 0] because that computing cyclic-distance could ensure the delta-coded bit range remains the same as encoded by the original quantized bits. The result of delta-coding on a filter-cluster in this example is formed of the first filter (b1) and delta-coded filters ({||*b*1−*b*2||, ||*b*2−*b*3||}).

For whole layer filters, we grouped *k* first filters in a matrix (called first filter matrix *D*^l*^), and *k* delta-coded filters in another matrix (called delta-coded filter matrix *D*^*l*^), and the detailed pseudocode for delta coding on a convolution layer is listed in Algorithm 2. At last, we separately apply Huffman coding to the first filter matrix and delta-coded filter matrix.

**Algorithm 2:** Delta coding on a convolution layer.

input: Quantized filter-clusters:$\left\{B_{1}^{l},\ldots ,B_{i}^{l},\ldots ,B_{L}^{l}\right\}$, quantized bit *q*^*l*^
Output: Delta-encoded form of *D*^l*^, *D*^*l*^
function Cyclic-distance(B^*^, *B*^*R*^, *r*)
for *M, w, h* in size of B^*^do
$B_{M,w,h}^{R}\leftarrow \min\left\{\left|\overset{*}{\underset{M,w,h}{B}}-B_{M,w,h}|,r-|B_{M,w,h}^{*}-B_{M,w,h}\right|\right\}$
return *B*^*R*^
for each Quantized filter-cluster: $B_{i}^{l}$ do
*j*←*j*+1
for each *j* from 1 to size($B_{i}^{l}$) do
$D^{l}\leftarrow \left\{D^{l},Cyclic-distance\left(b_{ij}^{l},b_{ij+1}^{l},2^{q^{l}}\right)\right\}$
return *D*^l*^, *D*^*l*^

For further compression, we observed that the order of filters in a filter-cluster could affect the compression rate due to the delta-coding step calculating the cyclic-distance on consecutive filters. Thus, by changing the order of filters in a filter-cluster, we could minimize the entropy of the delta-coded filter matrix. In this study, we take an easy example for explaining the effect of the permutation on filters. Suppose there exists 5 elements that index in [0, 2, 1, 3, 2]. When directly applying the delta coding, the delta-coded part will be [2, −1, 2, −1] whose entropy is 1.0 and the absolute distance is 6. However, it can lower the entropy by reordering the elements into [0, 1, 2, 2, 3] and then the delta-coded part is [1, 1, 0, 1], which gets 0.81 entropy and absolute distance is 3. According to the above idea, there comes a sub-problem : given a quantized filter-cluster $B_{i}^{l}$, the objective is to find the new order of filters in the given filter-cluster which makes the minimum total absolute cyclic-distance between the consecutive filters. The sub-problem can be viewed as the variant traveling salesman problems (TSP) (Lin and Kernighan, 1973). Different from the TSP problem which is to find a minimum Hamiltonian cycle, our sub-problem is to find the minimum Hamiltonian path which means that is no need to calculate the distance between the last node and the start node. It seems to slightly affect the total distance at the end; thus, for our subproblem, we applied a 2-approximation TSP algorithm for the permutation on filters in a quantized filter-cluster. At first, we generated the symmetric adjacency matrix in which each element represents the distance between filters. This symmetric adjacency matrix can be regarded as the weighted fully-connected graph G, and the filters are viewed as the nodes of graph G. Therefore, the problem can be converted into finding the minimum Hamiltonian path of graph G. Next, Kruskal's algorithm is utilized to find the minimum spanning three M of G and the Euler tour traversal for *M* is applied with the first filter in the given filter-cluster as the start point and then the Hamiltonian path in the traversal order was returned. The order of the Hamiltonian path is the new order for filters in the filter-cluster. In the end, the final compression model is obtained by directly recalling the delta and Huffman coding step introduced in the Algorithm 2 to these filter-clusters in the new orders of each convolution layer.

## 5. Experimental results

In this section, we provide experimental results by comparing our algorithm with other baselines and penalty sensitivity evaluation.

### 5.1. Experiment settings

In this section, we introduce the models for testing the compression and the baseline approaches with different experiment settings. For a fair comparison of compression with other baselines, the model accuracy after compression is given in Table 2.

**Table 2:** Accuracy (%) of different approaches.

	**CONV**	**CONV+Q+H**	**DeepC**	**OUR/P**	**OUR**
LeNet5	99.34	99.1	99.25	98.5	98.5
VGG (C10)	91.32	91.52	91.6	90.51	90.5

#### 5.1.1. Models and datasets

We test our algorithm on the well-known convolutional neural network models and datasets, including Lenet-5 on MNIST (Lenet5 for short) (LeCun et al., 1995) and VGG16 on CIFAR-10 [VGG (C10) for short] (Simonyan and Zisserman, 2014). Lenet-5 is a slight convolutional neural network, which has two convolution layers formed in 20 and 50 filters. In contrast, VGG16 is a deep and wide convolutional neural network, formed in 13 convolution layer, and the filter number ranges from 64 to 512. The MNIST dataset is a comprehensive set of 70,000 grayscale images of handwritten digits, widely acknowledged for benchmarking in machine learning algorithms. The images represent individual numerals ranging from 0 to 9, with each image dimension being 28× 28 pixels. The CIFAR-10 dataset encompasses 60,000 color images, each of 32× 32 pixels, distributed across 10 classes, ensuring an equal representation of 6,000 images per class.

#### 5.1.2. Baseline approaches

We compared our proposed algorithms (OUR) in Section 4 with other baselines. The baseline approaches are (i) the original convolutional neural network model (CONV), which does not apply any operation to compression of the model, stands for a base accuracy; (ii) the original convolution layer with quantization and Huffman coding (CONV+Q+H); (iii) deep compression (DeepC) (Han et al., 2015), one of the state-the-art model compression method; and (iv) the proposed algorithm without filter permutation (OUR/P). In the following, we will introduce the experiments set on different baselines in different models. For Lenet5 model compression, the algorithms which have pruning step are DeepC and OUR/P, and our algorithm OUR as well. Table 3 shows the pruning rate of these algorithms. For DeepC, we assigned the pruning rate according to the experiments in the proposed work. For OUR and OUR/P, filter-level pruning the whole filter lead to more accuracy sensitivity than threshold pruning in DeepC. Therefore, for pruning with an acceptable accuracy drop, the pruning rate of OUR and OUR/P are higher than DeepC. In addition, all the algorithms quantize each convolution layer to 3 bits for Lenet5. The specific hyper-parameter *k* of OUR and OUR/P is 2 for this model. For VGG (C10), we set quantization bit 5 according to the baseline DeepC. Again, the pruning rate for model VGG (C10) is shown in Table 4 and cluster number k of OUR and OUR/P is 4.

**Table 3:** Pruning rate (%) on Lenet5 of different approaches.

	**DeepC**	**OUR/P**	**OUR**
Conv1	66	65	65
Conv2	12	12	12

**Table 4:** Pruning rate (%) on VGG (C10) of different approaches.

	**DeepC**	**OUR/P**	**OUR**
Conv1	58	100	100
Conv2	22	100	100
Conv3	34	60	60
Conv4	36	60	60
Conv5	53	80	80
Conv6	24	40	40
Conv7	42	50	50
Conv8	32	40	40
Conv9	27	27	27
Conv10	34	34	34
Conv11	35	35	35
Conv12	29	29	29
Conv13	35	35	35

### 5.2. Compression rate comparison with baselines

**Figure 2:**
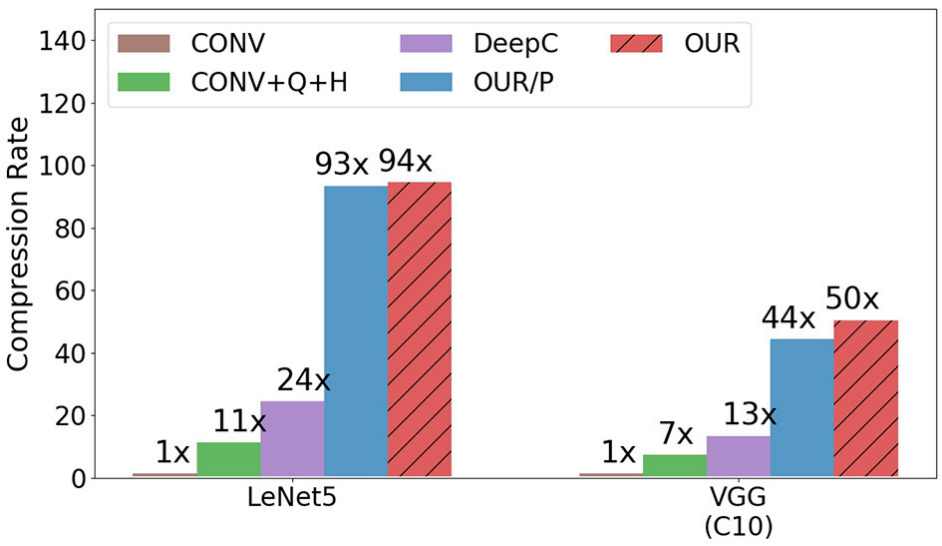
Compression rates of different approaches.

Figure 2 compares the compression rate of our proposed approach with other baselines. The compression rate is calculated as the memory size of the original parameter in convolution layers, divided by the memory size of the compression algorithm (including the codebook size and the filter-cluster assignment size if any). As shown in Figure 2, OUR/P and OUR outperform the other baselines. Especially, despite the higher pruning rate than DeepC on VGG (C10), our approach still achieve significant success in compression rate due to the great success of converting the distribution by delta coding similar filters. Table 5 gives a comparison of the entropy which explain the better compression rate for our algorithm.

**Table 5:** Layer-wise parameter entropy on VGG (C10).

	**Org. index**	**α = 0.05**	**α = 0.01**	**α = 0.02**
Conv1	4.41	4.76	4.81	4.66
Conv2	4.10	4.40	4.23	3.98
Conv3	4.39	4.78	4.75	4.62
Conv4	4.25	3.72	3.74	3.72
Conv5	4.24	3.77	3.74	3.76
Conv6	4.47	4.50	4.47	4.43
Conv7	4.24	2.84	2.83	2.79
Conv8	4.36	3.24	3.10	2.86
Conv9	4.30	1.91	1.62	1.23
Conv10	4.26	1.47	1.22	0.99
Conv11	4.07	1.09	0.79	0.63
Conv12	4.22	1.06	0.76	0.64
Conv13	4.05	0.71	0.52	0.45

CONV+Q+H only has a 7× compression rate on the VGG (C10) model and an 11× compression rate on the Lenet5 model. It is a very limited improvement on the compression rate for only applying quantization and Huffman coding. DeepC, the compression algorithm, proposed the compression pipeline for model compression. It achieves 13× on VGG (C10) model and 24× on the Lenet5 model. In the pruning step, DeepC achieves a 3× compress ton VGG (C10) model. It means that quantization and Huffman coding act an important role in the improvement of compression rate from 3× up to 13×. For the Lenet5 model, pruning compresses with a 7× compression rate, and after quantization and Huffman coding, the compression rate is up to 24×. Although quantization and Huffman coding seem to improve compression rates a lot in DeepC, it does not consider the best situation for Huffman coding compression. That is the reason that OUR significantly outperforms DeepC despite the higher pruning rate given in OUR. Therefore, OUR consider the best situation for Huffman coding to lead to a big success on the Lenet5 model and VGG (C10) model with 94× and 50× compression rate. For the VGG (C10) model, the filter-level pruning only compresses 2.8×. After the step for minimizing the filter-wise difference, the distribution of parameters tends to be more biased; if we directly apply quantization and Huffman coding without delta coding, it still can have a 21× compression rate higher than DeepC. With delta coding, make the compression rate twice times improvement due to the effect of low entropy for Huffman coding. However, for comparison on OUR/P and OUR, we found that the filter permutation could only slightly improve the compression rate on both models. The season for this situation is that the distance between filters is all similar, the way to permute the filter could only slightly reduce the total distance and also the entropy.

### 5.3. Penalty evaluation

To understand the effect of α, Table 4 shows the OUR/P compression approach makes different entropy with different α of each convolution layer on VGG (C10). The column Org. index stands for the original index value distribution, and the column is Delta-coded. The index stands for the distribution after delta coding with different α. Comparison with Org. index and Delta-coded. Index, the entropy significantly decreases with delta coding, as I mentioned in Section 5.2, Delta-coded. The index makes twice the compression than the Org. index. In this table, we can observe that our penalty have a great effect at the last seven convolution layer of the VGG (C10) model. It is reasonable that the first convolution layers are more likely to dominate the model accuracy. With larger α = 0.02, the entropy of the last layer reduces to 0.45 and the compression rate is 46× with 90.10% model accuracy. With smaller α = 0.005, the entropy of the last layer is 0.71 and is 40× with 90.23% accuracy. Therefore, the α is an important fact that affects the compression rate and trade-off between test accuracy and compression rate.

## 6. Conclusion

In this study the author proposed a new problem, called compression with filter-wise difference minimization which aims to minimize the difference between filters with an acceptable accuracy drop for further compression with Huffman coding and delta coding. The author proposed the algorithm to solve this problem and achieve significant compression rates, which are 94× of Lenet5 and 50× on VGG16. However, note that our current framework requires careful tuning of the penalty parameter to optimize the balance between the compression rate and test accuracy, which may pose a challenge for less experienced practitioners. In future, focus will be on enhancing the model compression methodology, specifically through the optimization of quantization bits. The author hypothesize that adjusting the quantization bit depth to suit individual filters will strike a more refined balance between model compression and performance. This line of research opens the door to making our compression method not only more efficient but also more adaptable to various problems.

## Data availability statement

Publicly available datasets were analyzed in this study. This data can be found at: http://yann.lecun.com/exdb/mnist/ and https://www.cs.toronto.edu/~kriz/cifar.html.

## Author contributions

All studies was completed by Y-TC.
